# Systematic cloning and analysis of autophagy-related genes from the silkworm *Bombyx mori*

**DOI:** 10.1186/1471-2199-10-50

**Published:** 2009-05-27

**Authors:** Xuan Zhang, Zhan-Ying Hu, Wei-Fang Li, Qing-Rong Li, Xiao-Juan Deng, Wan-Ying Yang, Yang Cao, Cong-Zhao Zhou

**Affiliations:** 1 Hefei National Laboratory for Physical Sciences at Microscale and School of Life Sciences, University of Science and Technology of China, Hefei Anhui 230027, PR China; 2 Department of Sericulture science, College of Animal Science, South China of Agricultural University, Guangzhou 510642, PR China

## Abstract

**Background:**

Through the whole life of eukaryotes, autophagy plays an important role in various biological events including development, differentiation and determination of lifespan. A full set of genes and their encoded proteins of this evolutionarily conserved pathway have been identified in many eukaryotic organisms from yeast to mammals. However, this pathway in the insect model organism, the silkworm *Bombyx mori*, remains poorly investigated.

**Results:**

Based on the autophagy pathway in several model organisms and a series of bioinformatic analyses, we have found more than 20 autophagy-related genes from the current database of the silkworm *Bombyx mori*. These genes could be further classified into the signal transduction pathway and two ubiquitin-like pathways. Using the mRNA extracted from the silkgland, we cloned the full length cDNA fragments of some key genes via reverse transcription PCR and 3' rapid amplification of cDNA ends (RACE). In addition, we found that the transcription levels of two indicator genes *BmATG8 *and *BmATG12 *in the silkgland tend to be increased from 1^st ^to 8^th ^day of the fifth instar larvae.

**Conclusion:**

Bioinformatics in combination with RT-PCR enable us to remodel a preliminary pathway of autophagy in the silkworm. Amplification and cloning of most autophagy-related genes from the silkgland indicated autophagy is indeed an activated process. Furthermore, the time-course transcriptional profiles of *BmATG8 *and *BmATG12 *revealed that both genes are up-regulated along the maturation of the silkgland during the fifth instar. These findings suggest that the autophagy should play an important role in *Bombyx mori *silkgland.

## Background

The programmed cell death (PCD) is a genetically regulated program of cell elimination, which is evolutionarily conserved in eukaryotes and plays a very important role in several physiological processes. PCD consists of two major types, apoptosis (type I) and autophagic cell death (type II) [1]. For a long time, autophagy has been described as a form of type II programmed cell death characterized by lysosomal activation and formation of autophagosomes. It is ubiquitous among eukaryotes functioning as a lysosome degradation pathway for recycling cytoplasmic materials especially long-lived proteins [2-4].

The formation of autophagosomes depends on the two ubiquitin-like conjugation systems, Atg8-PE (phosphatidylethanolamine) and Atg12-Atg5-Atg16 systems [5]. To trigger the former system, the C-terminal glycine residue of newly synthesized Atg8 has to be exposed by a cysteine-type endopeptidase Atg4. Subsequently, after being activated by an E1-like protein Atg7, Atg8 is conjugated to PE by a specific E2-like protein Atg3 [6]. In the latter system, Atg12 is activated by Atg7 and then transferred to an E2-like enzyme Atg10, via which conjugated to Atg5. Finally, the Atg12-Atg5 conjugate interacts with Atg16 to form a complex, which is localized to a membrane system to facilitate the maturation of autophagosomes [7-12].

There are several major signal transduction pathways and complexes involved in the induction of autophagy. One of these pathways is mediated by a serine/threonine kinase TOR (target of rapamycin), which takes part in most regulatory pathways in response to the changes in nutrient conditions and energy metabolism. TOR exerts an inhibitory effect on autophagy through various downstream effectors including Tap42 and P70s6 kinase (P70s6K) to regulate the transcription and/or translation of other related genes [13-18]. In addition to TOR, members in PtdIns 3-kinase class I (PI3K-I) and III (PI3K-III) also participate the regulation of autophagy. PI3K-I members phosphorylate PtdIns(4,5)P2 to produce PtdIns(3,4,5)P3, which binds to protein kinase B (Akt/Pkb) and its activator Pdk1 (phosphoinositide dependent kinase 1) [19-21]. The activation of PI3K-I/Akt pathway inhibits the GTPase-activating activity of Tsc2, leading to the relief of the inhibitory effect of Rheb-GDP on TOR/P70s6K signalling [22-26]. Mammalian hormones glucagon and insulin inhibit TOR by down-regulating PI3K-I, and so does ecdysone in *D. melanogaster*. The PI3K-I/Akt pathway is thought to down regulate the autophagy level, while PI3K-III, together with its membrane adaptor and autophagy protein Atg6 (Beclin1 in mammals), functions as an activator of autophagy and plays a crucial role in the early steps of autophagosome formation [27-32].

Recently, an explosion of studies on autophagy and cell survival indicates that autophagy may play an important role in the life cycle of eukaryotic organism. Autophagy may help cells to survive in death mutants, to crosstalk with the regulation of cell proliferation, to remove toxic cytoplasmic constituents, to reduce neurotoxicity of polyglutamine expansion proteins in some neurodegenerative diseases and also to be required for necrotic cell death. [33-38]. The metamorphic development from larvae to pupa is accompanying with the degeneration of specific larval tissues, such as the salivary glands of *Drosophila melanogaster *[39-41], intersegmental muscles [42], prothoracic glands [43] and silkglands of *B. mori *[44].

The silkgland is the largest tissue in the fifth and last instar of the silkworm *B. mori*. It consists of three parts: anterior (ASG), middle (MSG) and posterior silkgland (PSG)[45]. During the prepupal period, silkglands are degenerated via PCD pathway triggered by the steroid hormone ecdysone [44,46]. Both apoptotic and autophagic morphologies have been observed in the ASG during the larval-pupal metamorphosis, but the connections between them are still unclear [47-49]. The pupal differentiation of silkgland starts on the first day of the fifth instar and cell death in ASG is initiated on the third day [44]. Besides the PCD phenomenon found in ASG, we have also observed autophagic vacuolar formation in MSG during the prepupal period (unpublished data, Cao et al.). These findings indicate that autophagy may play a very important part in the differentiation and degeneration of silkgland, but the molecular mechanism remains unclear. Thus we performed a genome-wide search of autophagy-related genes in *B. mori*, aiming at remodelling the preliminary autophagy pathway for further systematic investigations.

## Results

### Identification of autophagy-related genes in *B. mori*

The autophagy-related genes from databases of *S. cerevisiae*, *D*. *melanogaster*, *A. mellifera *and *H. sapiens *genome were collected as queries. Using the blast website SilkwormBLAST to search against the Silkworm Genome Database (SilkDB), more than 20 autophagy-related genes could be identified as the top hits. However, even after a series of more sensitive profile-based searches, other members remain absent probably due to the low level of sequence homology. All hits of an E-value <1e-10 and alignment of >25% were collected and listed in Table 1. Most of these genes are involved in the two ubiquitin-like conjugation systems and the upstream signal transduction pathway. Besides, we also collected some important proteins taking part in the regulation of autophagy, including the protein serine/threonine kinase Atg1 [50], integral membrane protein Atg9 [51], phosphoinositide binding protein Atg18 [52] and the heat shock cognate protein HSC73 [53] (Table 1).

**Table 1:** Autophagy-related proteins identified from the SilkDB.

**Autophagy proteins**	***Bombyx mori *genes**	**Biochemical properties**
**Ubiquitin-like conjugation pathway**		
Atg3	*BGIBMGA003767-TA*	E2-like enzyme conjugates Atg8 to phosphatidylethanolamine (PE)
Atg4	*BGIBMGA004926-TA*	Cysteine protease, cleaves C-terminal extension or PE from Atg8
Atg5	*BGIBMGA007878-TA*	Conjugates to Atg12 through internal lysine
Atg7	*BGIBMGA001467-TA*	E1-like enzyme activates Atg8 and Atg12
Atg8	*BGIVMGA011783-PA*	Conjugates with Atg7, Atg3
Atg12	*BGIBMGA003954-TA*	Conjugates to Atg5 by E1 enzyme Atg7
Atg16	*BGIBMGA006504-TA*	Protein interacts with Atg12-Atg5
**Signal transduction pathway**		
PI3K-I	*BGIBMGA010561-TA*	Relieves the inhibitory effects on TOR through PI3K/Akt pathway
Pdk1	*BGIBMGA011755-TA*	The activator of Akt/Pkb
Akt/Pkb	*BGIBMGA014132-TA*	Protein kinase B, be activated by Pdk1
Tsc1	*BGIBMGA005845-TA*	Forms a complex with Tsc2
Tsc2	*BGIBMGA005686-TA*	Tuberous sclerosis complex 2, induce autophagy by inhibiting TOR
Rheb	*BGIBMGA006257-TA*	Be negatively regulated by TSC2 and positively regulates TOR
AMPK	*BGIBMGA013139-TA*	AMP activated protein kinase, stimulates autophagy by inhibiting TOR signal pathway
TOR	*BGIBMGA008952-TA*	Rapamycin target and a kinase involved in repression of autophagy
Tap42	*BGIBMGA013517-TA*	A target of the TOR kinases
Pp2A	*BGIBMGA010831-TA*	Protein phosphatase 2A, functions downstream of TOR
P70s6K	*BGIBMGA011088-TA*	A downstream effector of TOR, positively regulates autophagy
PI3K- III	*BGIBMGA007158-TA*	Contributes to the formation of autophagic vacuoles
Atg6	*BGIBMGA000092-TA*	Component of class III PI3K complexes
**others**		
Hsc73	*BGIBMGA002381-TA*	Involved in chaperone-mediated autophagy
Atg1	*BGIBMGA011986-TA*	Ser/Thr kinase, required for autophagy
Atg18	BGIBMGA007298-TA	Required for recycling of Atg9
Atg9	BGIBMGA012307-PA	Transmembrane protein, formation of CVT and autophagic vesicles

^a^Homologues were identified by a BLAST search against the Silkworm Genome Database (SilkDB).

^b^The alignment parameters were set as E-value of < 1e-10 and alignment of >25%.

### Remodelling the autophagy pathway

**Figure 1:**
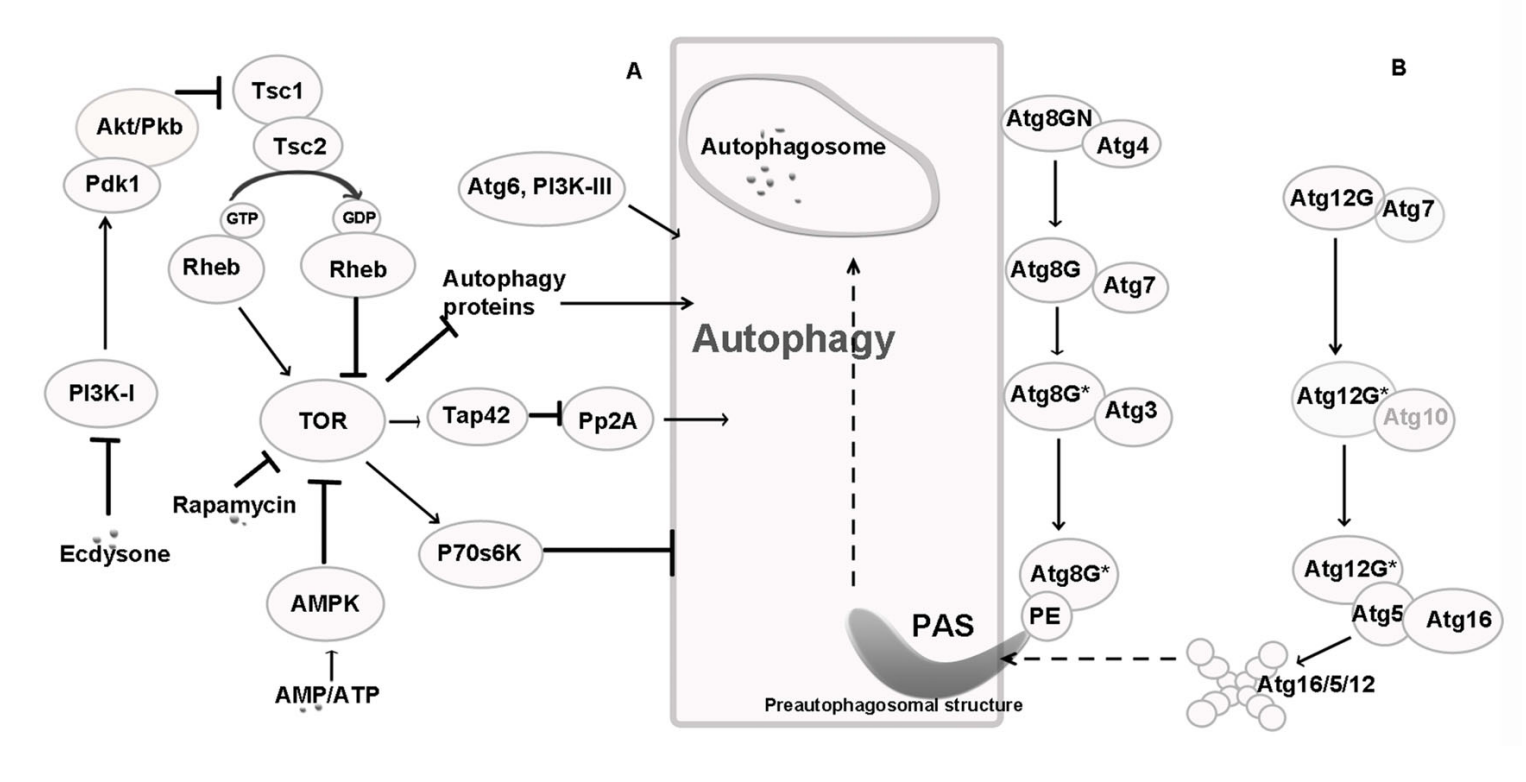
**Autophagy pathway in *Bombyx mori***. **A) **Signal transduction pathway. **B) **Two ubiquitin-like protein conjugation systems. The absent Atg10 is colored in grey.

Autophagy-related genes were first identified in yeast, and their homologs have recently been found and functionally characterized in higher eukaryotes. Evolutionary analyses indicated that autophagy is a highly conserved process among eukaryotes, such as yeast, plant, insect and mammal [54]. Based on the current knowledge, we remodel a preliminary autophagy pathway in *B. mori *(Figure 1). In the upstream signal transduction pathway, autophagic responses are developed by a series of kinases and phosphatases. TOR and PI3K are the major regulators in this signal transduction process (Figure 1A). In the Atg8-PE ubiquitin-like conjugation system, the last residue Asn117 of BmAtg8 is proposed to be cleaved by BmAtg4 for exposure of a C-terminal glycine residue. Then the modified BmAtg8 is activated by the E1-like enzyme BmAtg7 and finally transferred to PE by E2-like enzyme BmAtg3. In the Atg12-Atg5-Atg16 system, BmAtg12 should be activated by BmAtg7 to form a complex with BmAtg5. Subsequently, the BmAtg12-BmAtg5 conjugate interacts with BmAtg16, a protein containing a coiled-coil domain that mediates self-multimerization and the formation of an assembly of ~350-kDa (Figure 1B). All members in Atg12-Atg5-Atg16 conjugation pathway have been identified from the SilkDB except for Atg10 (colored in grey) (see the Discussion for more details).

### Most autophagy-related genes are actively transcribed in the silkgland

**Figure 2:**
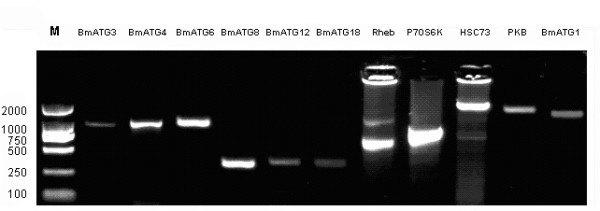
**Some autophagy-related genes in *Bombyx mori *amplified by PCR with specific primers**.

From the 6^th ^day through the fifth instar, both apoptotic and autophagic morphologies in the ASG [44] and MSG (unpublished data, Cao et al.) have been observed. We extracted total RNA from the silkglands of fifth instar larvae on the 3^rd ^day to validate these *in silico *identified genes by reverse-transcription PCR. Some representative PCR products were shown in Figure 2. *BmATG3*, *BmATG4*, *BmATG8 *and *BmATG12 *are genes involved in ubiquitin-like conjugation systems; *BmATG6*, *BmATG1 *and *BmAtg18 *are required for formation of autophagosomes; Homologs of *P70S6K, PKB *and *Rheb *are members of the upstream signal transduction pathways;*HSC73 *mediates the chaperone-mediated autophagy.

Furthermore, full-length cDNAs of *BmATG3*, *BmATG4*, *BmATG6, BmATG8 *and *BmATG12 *have been sequenced and deposited in GenBank under the accession numbers of FJ416327, FJ416326, FJ416328, FJ416330 and FJ416329, respectively. All proteins encoded by these genes are homogeneous to their orthologs in other eukaryotes (for the multialignments of Atg4, Atg6 and Atg5, see Additional file 1), indicating high conservation of the ubiquitin-like pathways in *B. mori*.

In the SilkDB, we found only a 5' fragment of putative *BmATG5*. To obtain its full-length cDNA, we used 3' RACE PCR method to amplify the unknown 3' flanking sequences. First-strand cDNA reverse-transcribed with an oligo(dT)-adaptor primer from total RNA was used as the template for 3' RACE PCR with gene specific primer GSP1 and out primer. The 3' RACE PCR product of *BmATG5 *was inserted into pGM-T vector for sequencing, and then this 3' segment was spliced with sequence of *BGIBMGA007878-TA *(SilkDB) to obtain the full-length cDNA sequence of *BmATG5*. The complete coding sequence of *BmATG5 *(amplified with full-length primers) has been deposited in GenBank under the accession number of FJ418152. Compared with the orthologs in other eukaryotes, BmAtg5 has the two highly conserved Atg16 binding domains (ubiquitin-like folds) and one Atg12 conjugation site (residue Lys149) (see Additional file 1).

Successful amplification of the coding sequences of some key autophagy-related genes listed in Table 1 indicated that they are indeed actively transcribed in the silkgland. Comparison to the orthologs further validated the high level of conservation of the two ubiquitin-like conjugation systems in the silkworm *B. mori*.

### Both BmATG8 and BmATG12 are up-regulated along the maturation of silkgland as revealed by semi-quantitative PCR

**Figure 3:**
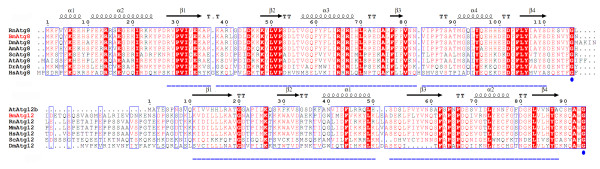
**Multiple alignments of A) Atg8 and B) Atg12 homologs**. The ubiquitin-like fold and the C-terminal glycine are marked in blue. All sequences were obtained from NCBI, SGD and SilkDB database, alignments were performed using the programs MultAlin [56] and ESPript [57]. Species abbreviations are *Bm *for *B. mori*, *Dm *for *D. melanogaster*, *Sc *for *S. cerevisiae*, *Rn *for *R. norvegicus*, *Mm *for *M. musculus*, *Hs *for *H. sapiens*, *At *for *A. thaliana, Am *for *A. mellifera *and *Dr *for *D. rerio*.

In attempt to monitor the expression pattern of some indicator genes in the silkgland at different stages of the fifth instar, we performed a time-course transcriptional profiling of *BmATG8 *and *BmATG12*. In mammalian cells, the protein levels of Atg5-Atg12 and/or LC3-II (Atg8 homologue) have been used as indicators for the autophagic activity [55]. Multiple alignments [56,57] indicated that both Atg8 and Atg12 homologs have an ubiquitin-like fold and a highly conserved glycine residue at the C-terminal region (Figure 3), which is essential for their conjugation to Atg3 and Atg5, respectively. Therefore, we used the transcription levels of *BmATG12 *and *BmATG8 *to indicate the autophagic activity in the silkgland of *B. mori*.

**Figure 4:**
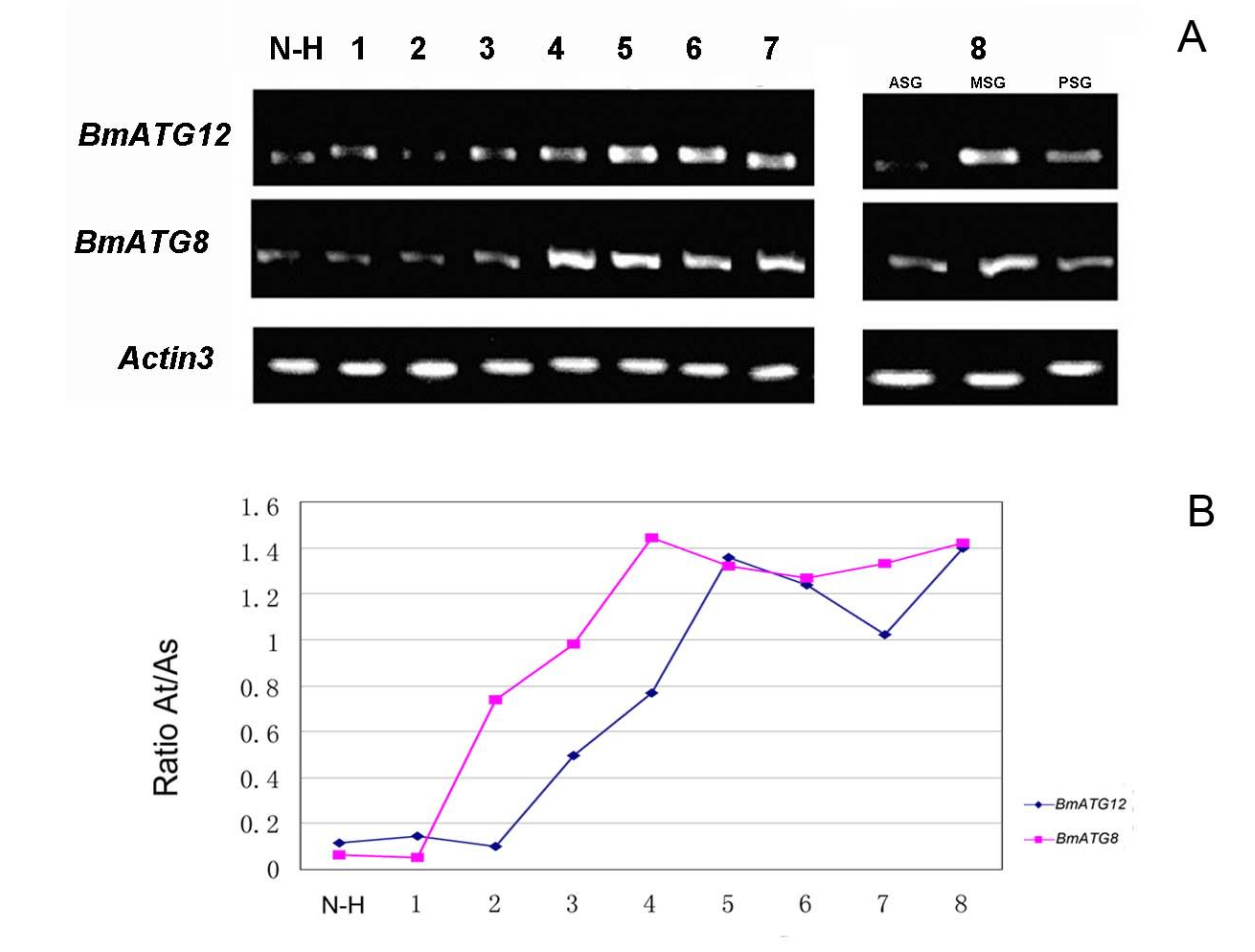
**Semi-quantitative PCR analyses of *BmATG8 *and *BmATG12***. **A) **Lane 1, Total RNA from newly hatched larva (N-H); Lane 2–8, Total RNA from silkgland of 1^st ^day to 7^th ^day fifth instar larvae (1–7); Lane 9–11, Total RNA from ASG(anterior silkgland), MSG(middle silkgland) and PSG(posterior silkgland) of the 8^th ^day fifth instar larvae. **B) **Quantification of the bands was performed by GIS 1D Software (Tanon, Shanghai, China) and the ratio of amplified target (At) to standard (As) was calculated for all samples.

Total RNA from the silkgland on each day in the fifth instar larvae was prepared for reverse-transcription. Double-stranded cDNA samples of the same quantity were used as templates for semi-quantitative PCR with primers for *BmATG8 *and *BmATG12*, respectively. The quantification of the bands was performed by GIS 1D Software (Tanon, Shanghai, China) and ratio of amplified target *BmATG8*/*BmATG12 *to standard *Actin3 *was calculated. As shown in Figure 4, the transcriptional levels of both *BmATG8 *and *BmATG12 *exhibit an overall tendency of increase from 1^st ^to 8^th ^day, and reach a plateau on the 4^th ^day (*BmATG8*) and the 5^th ^day (*BmATG12*) of fifth instar larvae, respectively. These results suggested a significant up-regulation of autophagic level in the silkgland of fifth instar larvae, which is in agreement with the previous histochemical investigations [47]. Thus we suggested that autophagy should take a crucial part in the differentiation and degeneration of the silkgland in prepupal of *B. mori*.

## Discussion

Along the metamorphic development from larvae to pupa, some larval organs of *B. mori *such as the larval midgut, intersegmental muscles and silkglands are degenerated and new imaginal structures are developed. The histolysis of these tissues was proposed to be achieved via programmed cell death and the degradation process in the ASG cells display both autophagic and apoptotic characteristics [44,46,58]. However, the *B. mori *genes or proteins required for autophagy have not been well characterized as those identified from the yeast or human [59].

In the present work, we performed a comprehensive bioinformatic analysis and found 24 autophagy-related proteins as the top hits from the SilkDB (Table 1). All homologs involved in the two ubiquitin-like pathways have been found in *B. mori *except for Atg10 which has not been identified from other two model insects, neither fruitfly nor honeybee. As well known, the E2-like enzyme Atg10 plays a very important part in transferring Atg12 to Atg5. It remains a puzzle that how the Atg12-Atg5-Atg16 conjugation system works without a functional Atg10. After performing a multiple alignment of the primary sequences of Atg10 against those of E2-like enzyme Atg3 from different species, we found that Atg3 proteins of some insects have low homology to mammalian Atg10. As shown in Additional file 2, both Atg3 and Atg10 have a highly conserved C-terminal catalytic cysteine (marked in blue) which is essential for the conjugation [60]. BmAtg3 might be able to recognize both BmAtg8 and BmAtg12 which share an ubiquitin-like fold and a highly conserved glycine residue at the C-terminal region (Figure 3). Thus we hypothesize that the function of Atg10 could be compensated by Atg3 in *B. mori *and other lepidoptera insects. Further in vivo experiments, especially identification of the E2-like enzyme of Atg12, are necessary to verify this hypothesis.

Based on these 24 genes and their high degree of conservation to the orthologs identified and functionally characterized in higher eukaryotes [4,61-63], a preliminary autophagy pathway in *B. mori *was remodelled (Figure 1). In addition to these *in silico *analyses, RT-PCR results strongly indicated that autophagy-related genes in ubiquitin-like conjugation systems are indeed actively transcribed in the silkgland. This is in agreement with the previous report that *B. mori *silkglands are committed to undergo PCD from the 3^rd ^day of the fifth instar and are degenerated completely soon after pupation [44]. To verify the temporal control of this process, we performed a time-course transcriptional profiling of two indicator genes *BmATG8 *and *BmATG12 *via semi-quantitative RT-PCR analysis. As expected, the transcriptional levels of both *BmATG8 *and *BmATG12 *exhibit a general tendency of increase from 1^st ^to 8^th ^day.

## Conclusion

As an evolutionarily conserved and finely regulated process, autophagy plays a very important role in various biological events during the whole life of eukaryotes, including the metamorphic development of *B. mori*. In the present work, we identified over 20 autophagy-related genes and remodelled the autophagy pathway in *B. mori*. Using the total RNA extracted from the silkgland of the fifth instar larvae on the 3^rd ^day as the initial template, the coding sequences of several key genes were further cloned by reverse transcription PCR and/or 3' RACE PCR experiments, indicating that most autophagy-related genes are actively transcribed in the silkgland. Furthermore, semi-quantitative PCR results indicated that both *BmATG8 *and *BmATG12 *are up-regulated in fifth instar larvae of *B. mori*. Taken together, our findings, in combination with the previous histochemical investigations, evidently suggest that autophagy executes a very important role in the differentiation and degeneration of silkgland during the metamorphosis from larva to pupa.

## Methods

### Bioinformatic analyses

All autophagy-related genes in yeast and homologs in *D. melanogaster*, *A. mellifera*, *H. sapiens, R. norvegicus*, *M. musculus*, *A. thaliana*, *A. mellifera*, and *D. rerio *were collected as queries for BLAST search against the silkworm database SilkDB, and then the top hits were selected for annotation. The databases used for sequence analyses include SGD, SilkBase, SilkDB, GenBank, FlyBase and SilkwormBLAST (Table 2). Full-length sequences of autophagy-related genes found from the SilkDB were listed in Table 1. The parameters for BLAST were set as E-value of < 1e-10 and alignment of >25%.

**Table 2:** Databases used for bioinformatic analysis and primers for PCR

**Databases **	
SGD	
SilkBase	
SilkDB	
GenBank	
FlyBase	
SilkwormBLAST	

**Primers**	

*BmATG8-FOR*	CATGCCATGGATGAAATTCCAATACAA
*BmATG8-REV*	GCGTCGACTTAATTTCCATAGACAT
*BmATG12-FOR*,	CATGCCATGGATGAGTGATGAGAAA
*BmATG12-REV*	GTGAATTCTCAGCCCCAAGCTTG
*Actin3-FOR*	AGAGGTTCCGTTGTCCCG,
*Actin3-REV*	GGCGGTGATCTCCTTCTGCA

^a^SGD, SilkBase, SilkDB, FlyBase and SilkwormBLAST were used for bioinformatic analysis, cDNA sequences were submitted to GenBank.

^b^The primers were used for semi-quantitative PCR analysis of *BmATG8 *and *BmATG12*.

### RNA extraction, cDNA preparation and PCR validation of some key genes

Total RNA extracted (Trizol, Invitrogen) from the silkglands of the fifth instar larvae at the 3^rd ^day was reverse-transcribed with Super Script III (Invitrogen). Primers were designed according to the sequences downloaded from SilkDB Database. The cDNA prepared from total RNA was used as template for PCR amplification in a volume of 25 μl with corresponding primers. The PCR products were separated on 1.5% agarose gel stained with GelRed.

The 3' end cDNA sequence of *BmATG5 *was obtained by using 3' RACE PCR with total RNA from mesenteron of *B. mori *in fifth instar. First strand cDNA was synthesized using an Oligo(dT)-Adaptor primer provided by Takara 3' RACE kit at 42°C for 60 min in a reaction volume of 10 μl. Then the cDNA was amplified using the 3' RACE Outer Primer (TACCGTCGTTCCACTAGTGATTT) and *BmATG5 *specific primer GSP1 (GGACTTTGACAGTACACTTC) as following procedure: 5 min at 94°C, 35 cycles of 30 sec at 94°C 30 sec at 55°C, 66 sec at 72°C, and then 10 min at 72°C. The PCR product was purified and cloned into pGM-T sequencing vector. According to the sequencing result, we spliced out the full-length sequence of *BmATG5 *and obtained it using PCR reaction with full-length primers.

### Semi-quantitative PCR analysis of BmATG8 and BmATG12

Total RNA from silkgland of each day in fifth instar larvae was reverse-transcribed to cDNA. The cDNAs of the same quantity was used as template for semi-quantitative PCR amplification in a volume of 20 μl with *BmATG8, BmATG12 *and *Actin3 *primers (Table 2). Each PCR reaction was performed by preheating the samples at 94°C for 5 min followed by 20 cycles of 94°C for 30 sec, 55°C for 30 sec and 72°C for 40 sec followed by an extra extension at 72°C for 10 min, the PCR products were separated on 1.5% agarose gel. Quantification of the bands was performed by GIS 1D Software (Tanon, Shanghai, China) and band intensities were expressed as relative absorbance units Volume (μg). The ratio of amplified *BmATG8 *and *BmATG12 *respectively to *Actin3 *was calculated for all samples.

## Authors' contributions

XZ performed the bioinformatic analyses, RNA extraction and RT-PCR, semi-quantitative PCR. YZH performed 3' RACE PCR of *BmATG5 *and obtained the full length cDNA sequence of some genes. CZZ and WFL coordinated the project and provided financial support. XZ and CZZ designed the experiments and wrote the paper. All authors have read and approved the final manuscript.

## Supplementary Material

Additional file 1 **Multiple alignments of A) Atg4, B) Atg6, and C) Atg5 homologs**. The catalytic residues of Atg4 are marked in blue. The conserved conjugation site to Atg12 in Atg5 is marked in blue, the ubiquitin-like folds UblA and UblB (marked with blue dashed line) are essential for the binding of Atg5 to Atg16. All sequences were obtained from NCBI, SGD and SilkDB database, alignments were performed using the programs MultAlin [56] and ESPript [57]. Species abbreviations are *Bm *for *B. mori*, *Dm *for *D. melanogaster*, *Sc *for *S. cerevisiae*, *Rn *for *R. norvegicus*, *Mm *for *M. musculus*, *Hs *for *H. sapiens*, *At *for *A. thaliana*, *Am *for *A. mellifera *and *Dr *for *D. rerio*. Click here for file

Additional file 2 **Multiple alignment of the primary sequences of Atg10 against Atg3 from different species**. The highly conserved catalytic cysteines of Atg3 homologs are marked in blue; The HR domain of Atg3 and the catalytic cysteine are marked with blue dashed line and dot, respectively. Species abbreviations are *Bm *for *B. mori*, *Am *for *A. mellifera*; *Dm *for *D. melanogaster*, *Ag *for *A. gambiae*, *Mm *for *M. musculus*, *Hs *for *H. sapiens*, *Rn *for *R. norvegicus*, *Sc *for *S. cerevisiae *and *At *for *A. thaliana*. Click here for file
