# Supplementary figures and images for: Systematic cloning and analysis of autophagy-related genes from the silkworm Bombyx mori
Source: BMC Mol Biol. 2009 May 27;10:50. doi: 10.1186/1471-2199-10-50 (PMC2693526; doi:10.1186/1471-2199-10-50)

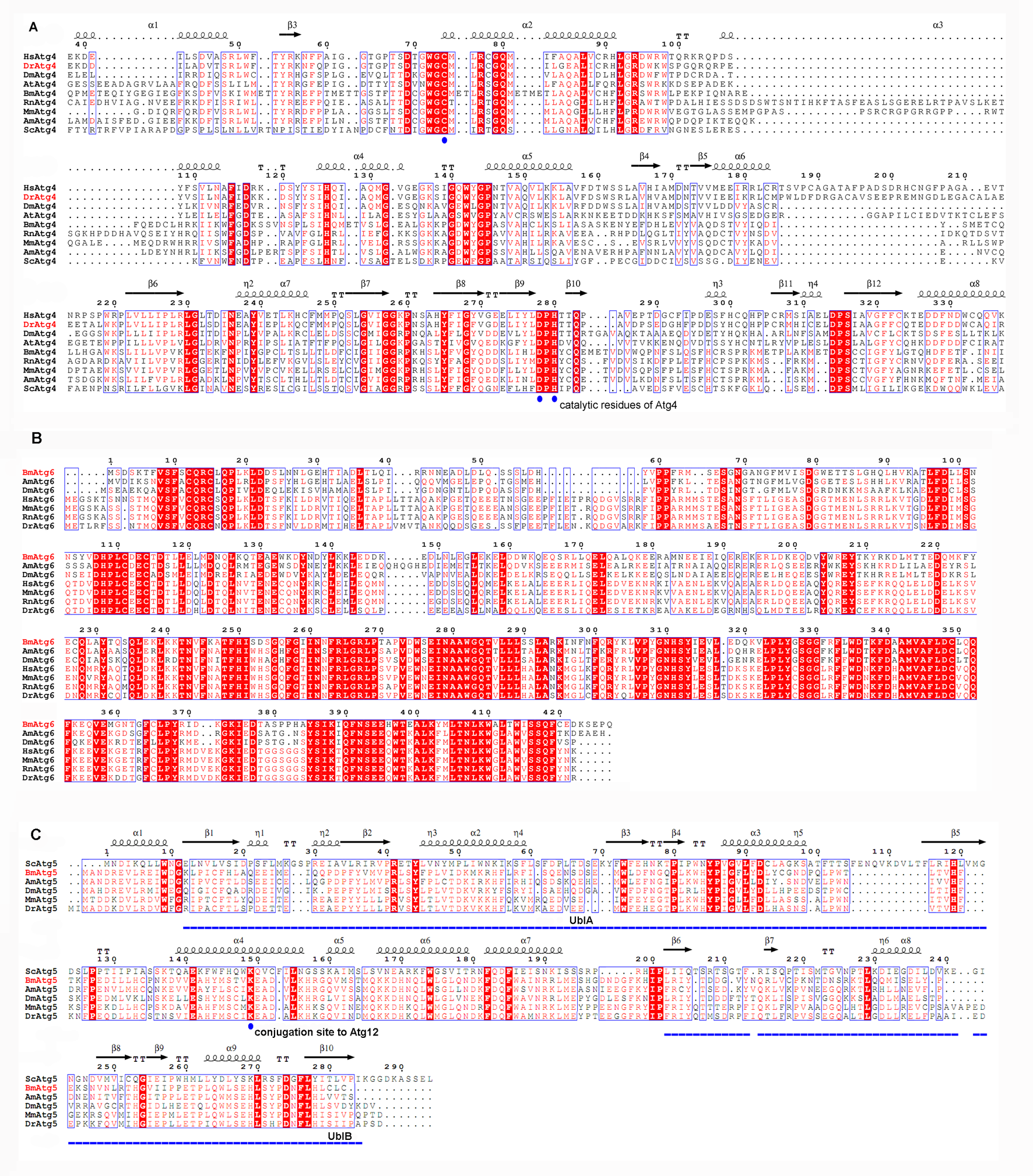

Supplement: Additional file 1 — Multiple alignments of A) Atg4, B) Atg6, and C) Atg5 homologs. The catalytic residues of Atg4 are marked in blue. The conserved conjugation site to Atg12 in Atg5 is marked in blue, the ubiquitin-like folds UblA and UblB (marked with blue dashed line) are essential for the binding of Atg5 to Atg16. All sequences were obtained from NCBI, SGD and SilkDB database, alignments were performed using the programs MultAlin [56] and ESPript [57]. Species abbreviations are Bm for B. mori, Dm for D. melanogaster, Sc for S. cerevisiae, Rn for R. norvegicus, Mm for M. musculus, Hs for H. sapiens, At for A. thaliana, Am for A. mellifera and Dr for D. rerio. [file 1471-2199-10-50-S1.tiff]

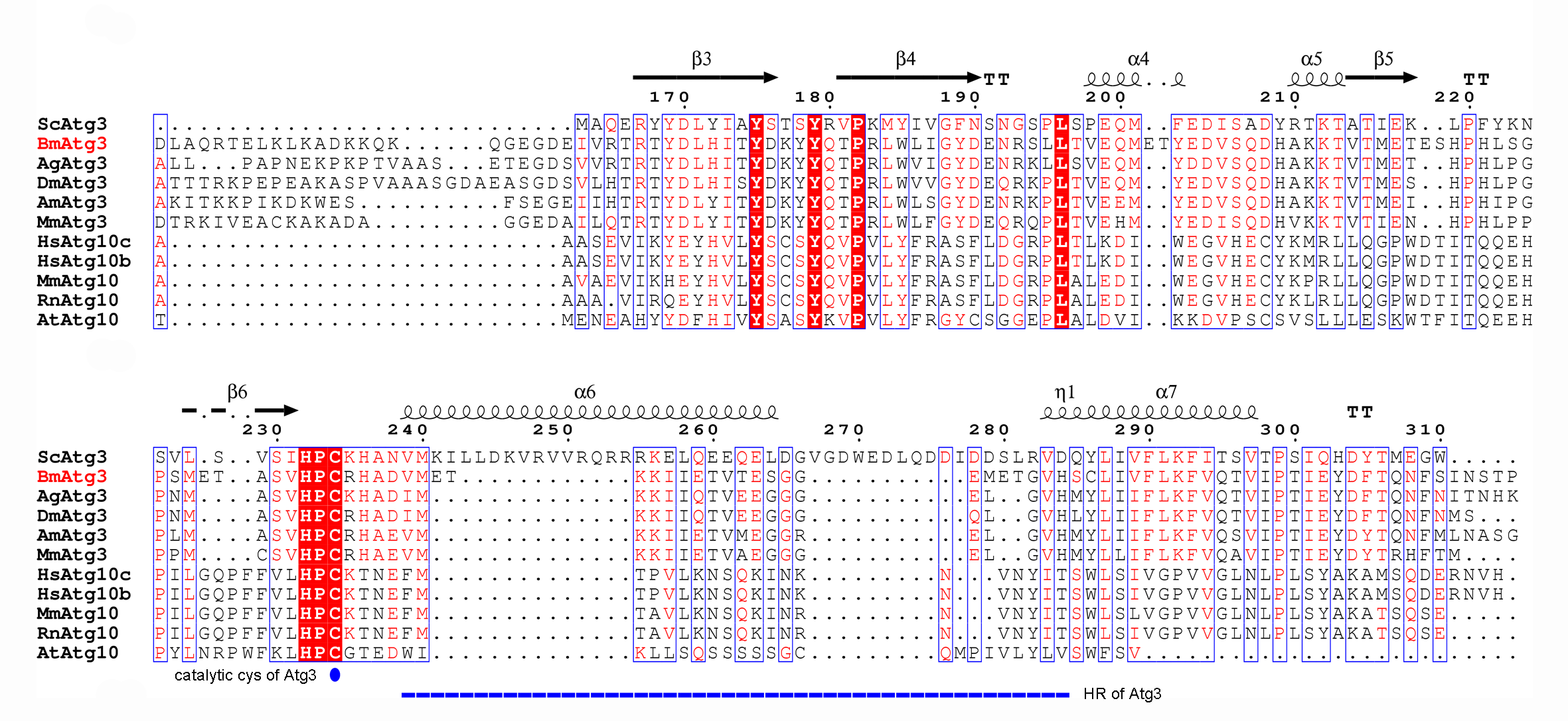

Supplement: Additional file 2 — Multiple alignment of the primary sequences of Atg10 against Atg3 from different species. The highly conserved catalytic cysteines of Atg3 homologs are marked in blue; The HR domain of Atg3 and the catalytic cysteine are marked with blue dashed line and dot, respectively. Species abbreviations are Bm for B. mori, Am for A. mellifera; Dm for D. melanogaster, Ag for A. gambiae, Mm for M. musculus, Hs for H. sapiens, Rn for R. norvegicus, Sc for S. cerevisiae and At for A. thaliana. [file 1471-2199-10-50-S2.tiff]
